# 1-Cyclo­propyl-2-(2-fluoro­phen­yl)-5-(4-fluoro­phen­yl)-3-phenyl­pentane-1,5-dione

**DOI:** 10.1107/S1600536813000032

**Published:** 2013-01-09

**Authors:** Thothadri Srinivasan, Govindaraj Senthilkumar, Haridoss Manikandan, Mannathusamy Gopalakrishanan, Devadasan Velmurugan

**Affiliations:** a Centre of Advanced Study in Crystallography and Biophysics, University of Madras, Guindy Campus, Chennai 600 025, India; b Department Of Chemistry, Annamalai University, Annamalainagar 608 002, Tamilnadu, India

## Abstract

In the title compound, C_26_H_22_F_2_O_2_, the cyclo­propane ring makes dihedral angles of 47.6 (2), 51.3 (2) and 63.9 (2)° with the 2-fluoro-substituted phenyl ring, the unsubstituted phenyl ring and the 4-fluoro-substituted phenyl ring, respectively. There is a short C—H⋯F contact in the molecule. In the crystal, weak C—H⋯F hydrogen bonds lead to chains of mol­ecules extending along the *b-*axis direction.

## Related literature

For the uses and biological importance of diketones, see: Bennett *et al.* (1999 ▶); Sato *et al.* (2008 ▶). For the crystal structure of a related compound, see: Li *et al.* (2008 ▶).
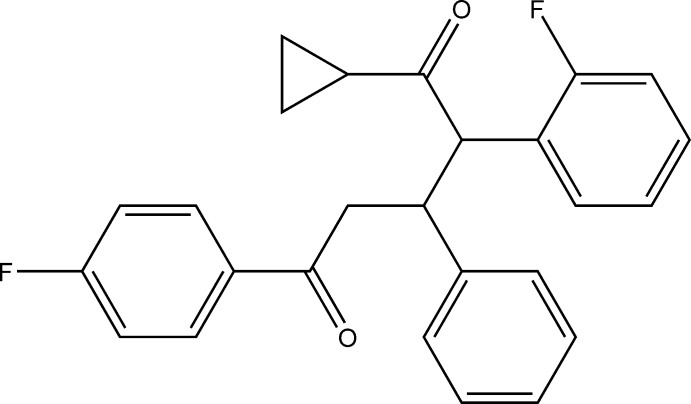


## Experimental

### 

#### Crystal data

C_26_H_22_F_2_O_2_
*M* *_r_* = 404.44
Monoclinic, 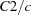

*a* = 38.9453 (14) Å
*b* = 5.7769 (2) Å
*c* = 18.3045 (7) Å
β = 95.334 (2)°
*V* = 4100.4 (3) Å^3^
*Z* = 8
Mo *K*α radiation
μ = 0.09 mm^−1^
*T* = 293 K
0.30 × 0.25 × 0.20 mm

#### Data collection

Bruker SMART APEXII area-detector diffractometer
Absorption correction: multi-scan (*SADABS*; Bruker, 2008 ▶) *T*_min_ = 0.972, *T*_max_ = 0.982
18721 measured reflections
5017 independent reflections
3326 reflections with *I* > 2σ(*I*)
*R*_int_ = 0.032

#### Refinement

*R*[*F* ^2^ > 2σ(*F* ^2^)] = 0.046
*wR*(*F* ^2^) = 0.133
*S* = 1.02
5017 reflections
271 parameters
H-atom parameters constrained
Δρ_max_ = 0.25 e Å^−3^
Δρ_min_ = −0.20 e Å^−3^

### 

Data collection: *APEX2* (Bruker, 2008 ▶); cell refinement: *SAINT* (Bruker, 2008 ▶); data reduction: *SAINT*; program(s) used to solve structure: *SHELXS97* (Sheldrick, 2008 ▶); program(s) used to refine structure: *SHELXL97* (Sheldrick, 2008 ▶); molecular graphics: *ORTEP-3* (Farrugia, 2012) ▶; software used to prepare material for publication: *SHELXL97* and *PLATON* (Spek, 2009 ▶).

## Supplementary Material

Crystal structure: contains datablock(s) global, I. DOI: 10.1107/S1600536813000032/pv2615sup1.cif

Structure factors: contains datablock(s) I. DOI: 10.1107/S1600536813000032/pv2615Isup2.hkl

Supplementary material file. DOI: 10.1107/S1600536813000032/pv2615Isup3.cml

Additional supplementary materials:  crystallographic information; 3D view; checkCIF report

## Figures and Tables

**Table 1:** Hydrogen-bond geometry (Å, °)

*D*—H⋯*A*	*D*—H	H⋯*A*	*D*⋯*A*	*D*—H⋯*A*
C12—H12⋯F1^i^	0.98	2.51	3.402 (2)	151
C5—H5⋯F1	0.98	2.42	2.833 (2)	105

Symmetry code: (i) 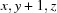.

**Fig. 1.**
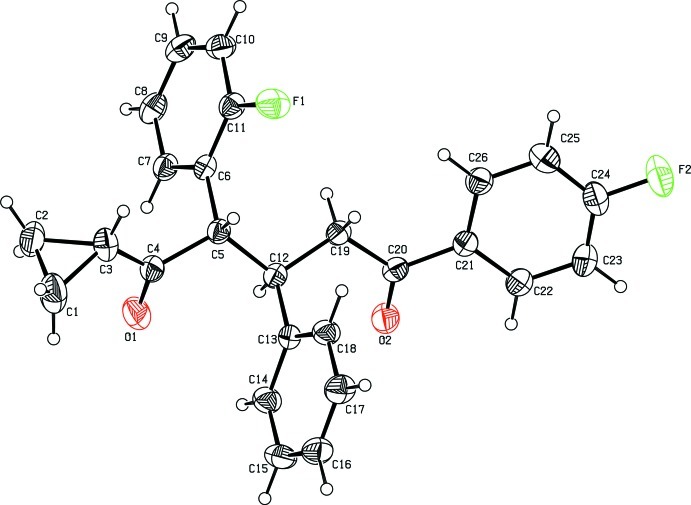
The molecular structure of the title compound, showing displacement ellipsoids drawn at the 30% probability level. H atoms are presented as small spheres of arbitrary radius.

**Fig. 2.**
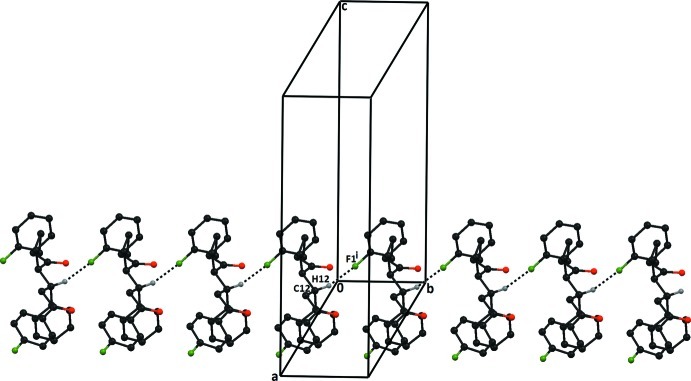
The crystal packing of the title compound viewed down b axis. H-atoms not involved in H-bonds have been excluded for clarity.

## supplementary crystallographic information

### Comment

Diketones are popular in organic synthesis for their applications in biology and medicine. They are known to exhibit antioxidants, antitumour and antibacterial activities (Bennett *et al.*,1999). They are also key intermediates in the preparation of various heterocyclic compounds (Sato *et al.*, 2008).

In the titled compound (Fig.1), the cyclopropane ring (C1/C2/C3) makes a dihedral angle of 47.6 (2)° with the fluoro substituted phenyl ring (C6/C7/C8/C9/C10/C11). It makes a dihedral angle of 51.3 (2)° with the unsubstituted phenyl ring (C13/C14/C15/C16/C17/C18) and a dihedral angle of 63.9 (2)° with the fluoro substituted phenyl ring (C21/C22/C23/C24/C25/C26). The dihedral angle between the unsubstituted phenyl ring and the fluoro substituted phenyl ring (C6–C11) is 3.86 (9)° and the dihedral angle between the unsubstituted phenyl ring and the fluoro substituted phenyl ring (C21–C26) is 69.43 (9)°. The dihedral angle between the two fluoro substituted phenyl rings is 67.28 (9)°. The packing of the crystal is stabilized by weak C–H···F hydrogen bonding interactions (Tab. 1 & Fig. 2).

### Experimental

A mixture of 4-fluoroacetophenone (0.01 mole), benzaldehyde (0.01 mole), cyclopropyl 2-fluorobenzyl ketone (0.01 mole) and sodium hydroxide solution (10 ml, 10%) in ethanol (50 ml) was stirred for 3 hrs at room temperature. The solid that separated was filtered and washed with distilled water. The product was recrystallised from ethanol to yield the crystals of the title compound suitable for X-ray crystallographic studies. Yield = 97%, melting point = 391–393 K.

### Refinement

All H-atoms were positioned and refined using a riding model with C–H = 0.98, 0.97 and 0.93 Å for methine, methylelne and aryl H-atoms, respectively. The H-atoms were allowed *U*_iso_ = 1.2*U*_eq_(C).

### Crystal data

**Table:**

C_26_H_22_F_2_O_2_	*F*(000) = 1696
*M**_r_* = 404.44	*D*_x_ = 1.310 Mg m^−^^3^
Monoclinic, *C*2/*c*	Mo *K*α radiation, λ = 0.71073 Å
Hall symbol: -C 2yc	Cell parameters from 5017 reflections
*a* = 38.9453 (14) Å	θ = 1.1–28.3°
*b* = 5.7769 (2) Å	µ = 0.09 mm^−^^1^
*c* = 18.3045 (7) Å	*T* = 293 K
β = 95.334 (2)°	Block, colourless
*V* = 4100.4 (3) Å^3^	0.30 × 0.25 × 0.20 mm
*Z* = 8	

### Data collection

**Table:**

Bruker SMART APEXII area-detector diffractometer	5017 independent reflections
Radiation source: fine-focus sealed tube	3326 reflections with *I* > 2σ(*I*)
Graphite monochromator	*R*_int_ = 0.032
ω and φ scans	θ_max_ = 28.3°, θ_min_ = 1.1°
Absorption correction: multi-scan (*SADABS*; Bruker, 2008)	*h* = −50→43
*T*_min_ = 0.972, *T*_max_ = 0.982	*k* = −7→7
18721 measured reflections	*l* = −24→24

### Refinement

**Table:**

Refinement on *F*^2^	Primary atom site location: structure-invariant direct methods
Least-squares matrix: full	Secondary atom site location: difference Fourier map
*R*[*F*^2^ > 2σ(*F*^2^)] = 0.046	Hydrogen site location: inferred from neighbouring sites
*wR*(*F*^2^) = 0.133	H-atom parameters constrained
*S* = 1.02	*w* = 1/[σ^2^(*F*_o_^2^) + (0.0504*P*)^2^ + 2.5291*P*] where *P* = (*F*_o_^2^ + 2*F*_c_^2^)/3
5017 reflections	(Δ/σ)_max_ < 0.001
271 parameters	Δρ_max_ = 0.25 e Å^−^^3^
0 restraints	Δρ_min_ = −0.20 e Å^−^^3^

### Special details

**Table:**

Geometry. All esds (except the esd in the dihedral angle between two l.s. planes) are estimated using the full covariance matrix. The cell esds are taken into account individually in the estimation of esds in distances, angles and torsion angles; correlations between esds in cell parameters are only used when they are defined by crystal symmetry. An approximate (isotropic) treatment of cell esds is used for estimating esds involving l.s. planes.
Refinement. Refinement of F^2^ against ALL reflections. The weighted R-factor wR and goodness of fit S are based on F^2^, conventional R-factors R are based on F, with F set to zero for negative F^2^. The threshold expression of F^2^ > 2sigma(F^2^) is used only for calculating R-factors(gt) etc. and is not relevant to the choice of reflections for refinement. R-factors based on F^2^ are statistically about twice as large as those based on F, and R- factors based on ALL data will be even larger.

### Fractional atomic coordinates and isotropic or equivalent isotropic displacement parameters (Å^2^)

**Table:**

	*x*	*y*	*z*	*U*_iso_*/*U*_eq_	
C1	0.00262 (5)	0.6122 (5)	0.08432 (12)	0.0835 (7)	
H1A	−0.0048	0.7524	0.0583	0.100*	
H1B	−0.0123	0.4795	0.0741	0.100*	
C2	0.01946 (5)	0.6368 (5)	0.15898 (11)	0.0768 (6)	
H2A	0.0149	0.5191	0.1946	0.092*	
H2B	0.0224	0.7920	0.1788	0.092*	
C3	0.04098 (5)	0.5684 (4)	0.09772 (11)	0.0628 (5)	
H3	0.0487	0.4070	0.0966	0.075*	
C4	0.06452 (4)	0.7452 (3)	0.07330 (9)	0.0494 (4)	
C5	0.10063 (4)	0.6636 (3)	0.05999 (8)	0.0402 (3)	
H5	0.0987	0.5069	0.0395	0.048*	
C6	0.12138 (4)	0.6493 (3)	0.13465 (8)	0.0403 (3)	
C7	0.12022 (4)	0.8227 (3)	0.18717 (8)	0.0473 (4)	
H7	0.1065	0.9523	0.1765	0.057*	
C8	0.13904 (5)	0.8064 (3)	0.25495 (9)	0.0553 (4)	
H8	0.1378	0.9238	0.2894	0.066*	
C9	0.15958 (5)	0.6159 (3)	0.27130 (9)	0.0565 (5)	
H9	0.1721	0.6045	0.3170	0.068*	
C10	0.16176 (5)	0.4425 (3)	0.22048 (10)	0.0549 (4)	
H10	0.1758	0.3142	0.2310	0.066*	
C11	0.14271 (4)	0.4636 (3)	0.15372 (9)	0.0464 (4)	
C12	0.11779 (4)	0.8197 (3)	0.00456 (8)	0.0406 (3)	
H12	0.1183	0.9786	0.0233	0.049*	
C13	0.09715 (4)	0.8184 (3)	−0.07003 (8)	0.0397 (3)	
C14	0.07706 (5)	1.0070 (3)	−0.09310 (10)	0.0536 (4)	
H14	0.0760	1.1340	−0.0622	0.064*	
C15	0.05858 (5)	1.0098 (4)	−0.16130 (11)	0.0645 (5)	
H15	0.0451	1.1378	−0.1757	0.077*	
C16	0.05997 (5)	0.8243 (4)	−0.20788 (10)	0.0615 (5)	
H16	0.0476	0.8265	−0.2539	0.074*	
C17	0.07965 (5)	0.6367 (3)	−0.18606 (10)	0.0596 (5)	
H17	0.0807	0.5106	−0.2174	0.072*	
C18	0.09808 (5)	0.6331 (3)	−0.11760 (9)	0.0506 (4)	
H18	0.1113	0.5039	−0.1034	0.061*	
C19	0.15504 (4)	0.7407 (3)	−0.00056 (9)	0.0468 (4)	
H19A	0.1669	0.7392	0.0484	0.056*	
H19B	0.1548	0.5833	−0.0190	0.056*	
C20	0.17512 (4)	0.8902 (3)	−0.04919 (8)	0.0436 (4)	
C21	0.20585 (4)	0.7867 (3)	−0.08010 (8)	0.0417 (3)	
C22	0.22127 (5)	0.9066 (3)	−0.13411 (10)	0.0552 (4)	
H22	0.2121	1.0477	−0.1507	0.066*	
C23	0.24992 (5)	0.8207 (4)	−0.16359 (11)	0.0689 (6)	
H23	0.2600	0.9015	−0.2000	0.083*	
C24	0.26322 (5)	0.6147 (4)	−0.13818 (11)	0.0625 (5)	
C25	0.24916 (5)	0.4901 (3)	−0.08487 (11)	0.0613 (5)	
H25	0.2589	0.3506	−0.0682	0.074*	
C26	0.22017 (4)	0.5761 (3)	−0.05628 (10)	0.0520 (4)	
H26	0.2101	0.4921	−0.0206	0.062*	
O1	0.05644 (4)	0.9477 (3)	0.06815 (9)	0.0757 (4)	
O2	0.16725 (3)	1.0902 (2)	−0.06242 (7)	0.0608 (3)	
F1	0.14564 (3)	0.29321 (18)	0.10349 (6)	0.0686 (3)	
F2	0.29148 (3)	0.5295 (3)	−0.16639 (8)	0.0974 (5)	

### Atomic displacement parameters (Å^2^)

**Table:**

	*U*^11^	*U*^22^	*U*^33^	*U*^12^	*U*^13^	*U*^23^
C1	0.0478 (11)	0.132 (2)	0.0718 (13)	−0.0092 (12)	0.0112 (10)	−0.0214 (14)
C2	0.0593 (12)	0.1175 (19)	0.0566 (11)	0.0007 (12)	0.0211 (10)	0.0027 (12)
C3	0.0491 (10)	0.0742 (13)	0.0678 (12)	−0.0008 (9)	0.0193 (9)	−0.0031 (10)
C4	0.0478 (9)	0.0616 (11)	0.0393 (8)	0.0067 (8)	0.0073 (7)	−0.0017 (8)
C5	0.0431 (8)	0.0415 (8)	0.0367 (7)	0.0007 (7)	0.0072 (6)	−0.0015 (6)
C6	0.0438 (8)	0.0432 (8)	0.0350 (7)	−0.0025 (7)	0.0096 (6)	0.0018 (6)
C7	0.0551 (10)	0.0476 (9)	0.0403 (8)	0.0012 (8)	0.0107 (7)	−0.0014 (7)
C8	0.0668 (11)	0.0597 (11)	0.0402 (8)	−0.0128 (9)	0.0092 (8)	−0.0061 (8)
C9	0.0608 (11)	0.0682 (12)	0.0395 (8)	−0.0144 (9)	−0.0011 (8)	0.0081 (8)
C10	0.0562 (10)	0.0533 (10)	0.0542 (10)	0.0009 (8)	0.0002 (8)	0.0125 (8)
C11	0.0545 (10)	0.0425 (9)	0.0428 (8)	−0.0013 (7)	0.0073 (7)	−0.0007 (7)
C12	0.0445 (8)	0.0402 (8)	0.0377 (7)	−0.0009 (7)	0.0071 (6)	0.0000 (6)
C13	0.0393 (8)	0.0422 (8)	0.0383 (7)	0.0015 (6)	0.0080 (6)	0.0014 (6)
C14	0.0599 (11)	0.0474 (9)	0.0532 (10)	0.0121 (8)	0.0037 (8)	−0.0021 (8)
C15	0.0638 (12)	0.0655 (12)	0.0625 (11)	0.0212 (10)	−0.0038 (10)	0.0098 (10)
C16	0.0576 (11)	0.0767 (13)	0.0478 (9)	0.0017 (10)	−0.0072 (8)	0.0041 (9)
C17	0.0727 (12)	0.0582 (11)	0.0468 (9)	0.0041 (9)	−0.0006 (9)	−0.0088 (8)
C18	0.0595 (10)	0.0470 (9)	0.0450 (9)	0.0119 (8)	0.0027 (8)	−0.0011 (7)
C19	0.0426 (8)	0.0560 (10)	0.0420 (8)	−0.0002 (7)	0.0046 (7)	0.0090 (7)
C20	0.0448 (9)	0.0466 (9)	0.0389 (8)	−0.0052 (7)	0.0013 (7)	0.0029 (7)
C21	0.0393 (8)	0.0467 (9)	0.0386 (7)	−0.0071 (7)	0.0009 (6)	−0.0019 (7)
C22	0.0538 (10)	0.0564 (10)	0.0564 (10)	−0.0007 (8)	0.0111 (8)	0.0107 (8)
C23	0.0596 (12)	0.0859 (15)	0.0649 (12)	−0.0009 (11)	0.0246 (10)	0.0106 (11)
C24	0.0458 (10)	0.0771 (13)	0.0657 (12)	0.0027 (9)	0.0114 (9)	−0.0142 (10)
C25	0.0554 (11)	0.0542 (10)	0.0735 (12)	0.0065 (9)	0.0021 (10)	−0.0025 (10)
C26	0.0503 (10)	0.0512 (10)	0.0548 (10)	−0.0027 (8)	0.0061 (8)	0.0052 (8)
O1	0.0719 (9)	0.0683 (9)	0.0911 (11)	0.0222 (7)	0.0290 (8)	0.0081 (8)
O2	0.0657 (8)	0.0478 (7)	0.0717 (8)	0.0030 (6)	0.0217 (7)	0.0074 (6)
F1	0.0890 (8)	0.0519 (6)	0.0636 (6)	0.0169 (6)	0.0003 (6)	−0.0103 (5)
F2	0.0719 (8)	0.1137 (11)	0.1122 (11)	0.0203 (7)	0.0387 (8)	−0.0110 (9)

### Geometric parameters (Å, º)

**Table:**

C1—C2	1.467 (3)	C13—C18	1.382 (2)
C1—C3	1.513 (3)	C13—C14	1.384 (2)
C1—H1A	0.9700	C14—C15	1.382 (3)
C1—H1B	0.9700	C14—H14	0.9300
C2—C3	1.514 (3)	C15—C16	1.373 (3)
C2—H2A	0.9700	C15—H15	0.9300
C2—H2B	0.9700	C16—C17	1.366 (3)
C3—C4	1.469 (3)	C16—H16	0.9300
C3—H3	0.9800	C17—C18	1.385 (2)
C4—O1	1.213 (2)	C17—H17	0.9300
C4—C5	1.524 (2)	C18—H18	0.9300
C5—C6	1.524 (2)	C19—C20	1.510 (2)
C5—C12	1.554 (2)	C19—H19A	0.9700
C5—H5	0.9800	C19—H19B	0.9700
C6—C11	1.382 (2)	C20—O2	1.2138 (19)
C6—C7	1.392 (2)	C20—C21	1.495 (2)
C7—C8	1.384 (2)	C21—C22	1.389 (2)
C7—H7	0.9300	C21—C26	1.391 (2)
C8—C9	1.377 (3)	C22—C23	1.376 (3)
C8—H8	0.9300	C22—H22	0.9300
C9—C10	1.375 (3)	C23—C24	1.362 (3)
C9—H9	0.9300	C23—H23	0.9300
C10—C11	1.375 (2)	C24—F2	1.351 (2)
C10—H10	0.9300	C24—C25	1.367 (3)
C11—F1	1.3589 (18)	C25—C26	1.380 (3)
C12—C13	1.519 (2)	C25—H25	0.9300
C12—C19	1.532 (2)	C26—H26	0.9300
C12—H12	0.9800		
			
C2—C1—C3	61.04 (13)	C13—C12—H12	108.1
C2—C1—H1A	117.7	C19—C12—H12	108.1
C3—C1—H1A	117.7	C5—C12—H12	108.1
C2—C1—H1B	117.7	C18—C13—C14	117.68 (15)
C3—C1—H1B	117.7	C18—C13—C12	121.82 (14)
H1A—C1—H1B	114.8	C14—C13—C12	120.49 (14)
C1—C2—C3	60.98 (13)	C15—C14—C13	121.11 (16)
C1—C2—H2A	117.7	C15—C14—H14	119.4
C3—C2—H2A	117.7	C13—C14—H14	119.4
C1—C2—H2B	117.7	C16—C15—C14	120.31 (17)
C3—C2—H2B	117.7	C16—C15—H15	119.8
H2A—C2—H2B	114.8	C14—C15—H15	119.8
C4—C3—C1	117.96 (19)	C17—C16—C15	119.41 (17)
C4—C3—C2	116.61 (18)	C17—C16—H16	120.3
C1—C3—C2	57.98 (13)	C15—C16—H16	120.3
C4—C3—H3	117.1	C16—C17—C18	120.40 (17)
C1—C3—H3	117.1	C16—C17—H17	119.8
C2—C3—H3	117.1	C18—C17—H17	119.8
O1—C4—C3	121.98 (17)	C13—C18—C17	121.09 (16)
O1—C4—C5	121.47 (16)	C13—C18—H18	119.5
C3—C4—C5	116.41 (15)	C17—C18—H18	119.5
C6—C5—C4	107.08 (12)	C20—C19—C12	114.29 (13)
C6—C5—C12	113.11 (12)	C20—C19—H19A	108.7
C4—C5—C12	112.61 (13)	C12—C19—H19A	108.7
C6—C5—H5	107.9	C20—C19—H19B	108.7
C4—C5—H5	107.9	C12—C19—H19B	108.7
C12—C5—H5	107.9	H19A—C19—H19B	107.6
C11—C6—C7	116.21 (14)	O2—C20—C21	120.10 (14)
C11—C6—C5	121.66 (13)	O2—C20—C19	121.79 (15)
C7—C6—C5	122.13 (14)	C21—C20—C19	118.10 (14)
C8—C7—C6	121.43 (16)	C22—C21—C26	118.17 (16)
C8—C7—H7	119.3	C22—C21—C20	118.95 (15)
C6—C7—H7	119.3	C26—C21—C20	122.88 (14)
C9—C8—C7	119.85 (16)	C23—C22—C21	121.29 (17)
C9—C8—H8	120.1	C23—C22—H22	119.4
C7—C8—H8	120.1	C21—C22—H22	119.4
C10—C9—C8	120.41 (16)	C24—C23—C22	118.51 (18)
C10—C9—H9	119.8	C24—C23—H23	120.7
C8—C9—H9	119.8	C22—C23—H23	120.7
C11—C10—C9	118.35 (17)	F2—C24—C23	119.03 (19)
C11—C10—H10	120.8	F2—C24—C25	118.37 (19)
C9—C10—H10	120.8	C23—C24—C25	122.60 (18)
F1—C11—C10	117.72 (15)	C24—C25—C26	118.49 (18)
F1—C11—C6	118.55 (14)	C24—C25—H25	120.8
C10—C11—C6	123.72 (15)	C26—C25—H25	120.8
C13—C12—C19	111.64 (12)	C25—C26—C21	120.94 (17)
C13—C12—C5	111.11 (12)	C25—C26—H26	119.5
C19—C12—C5	109.56 (12)	C21—C26—H26	119.5
			
C2—C1—C3—C4	−105.4 (2)	C5—C12—C13—C18	−76.16 (18)
C1—C2—C3—C4	107.8 (2)	C19—C12—C13—C14	−132.39 (16)
C1—C3—C4—O1	27.0 (3)	C5—C12—C13—C14	104.98 (17)
C2—C3—C4—O1	−39.1 (3)	C18—C13—C14—C15	0.0 (3)
C1—C3—C4—C5	−157.23 (16)	C12—C13—C14—C15	178.94 (16)
C2—C3—C4—C5	136.69 (17)	C13—C14—C15—C16	−0.3 (3)
O1—C4—C5—C6	95.68 (19)	C14—C15—C16—C17	0.3 (3)
C3—C4—C5—C6	−80.10 (17)	C15—C16—C17—C18	0.0 (3)
O1—C4—C5—C12	−29.3 (2)	C14—C13—C18—C17	0.3 (3)
C3—C4—C5—C12	154.95 (14)	C12—C13—C18—C17	−178.64 (16)
C4—C5—C6—C11	136.23 (16)	C16—C17—C18—C13	−0.3 (3)
C12—C5—C6—C11	−99.12 (17)	C13—C12—C19—C20	60.36 (18)
C4—C5—C6—C7	−44.37 (19)	C5—C12—C19—C20	−176.12 (13)
C12—C5—C6—C7	80.28 (17)	C12—C19—C20—O2	23.4 (2)
C11—C6—C7—C8	−1.0 (2)	C12—C19—C20—C21	−157.76 (14)
C5—C6—C7—C8	179.55 (15)	O2—C20—C21—C22	−11.6 (2)
C6—C7—C8—C9	0.3 (3)	C19—C20—C21—C22	169.58 (15)
C7—C8—C9—C10	0.6 (3)	O2—C20—C21—C26	167.32 (16)
C8—C9—C10—C11	−0.6 (3)	C19—C20—C21—C26	−11.5 (2)
C9—C10—C11—F1	178.71 (15)	C26—C21—C22—C23	0.0 (3)
C9—C10—C11—C6	−0.1 (3)	C20—C21—C22—C23	179.00 (17)
C7—C6—C11—F1	−177.89 (14)	C21—C22—C23—C24	−0.5 (3)
C5—C6—C11—F1	1.5 (2)	C22—C23—C24—F2	−179.57 (18)
C7—C6—C11—C10	0.9 (2)	C22—C23—C24—C25	0.2 (3)
C5—C6—C11—C10	−179.62 (15)	F2—C24—C25—C26	−179.61 (17)
C6—C5—C12—C13	177.05 (12)	C23—C24—C25—C26	0.6 (3)
C4—C5—C12—C13	−61.37 (16)	C24—C25—C26—C21	−1.1 (3)
C6—C5—C12—C19	53.22 (17)	C22—C21—C26—C25	0.8 (2)
C4—C5—C12—C19	174.80 (13)	C20—C21—C26—C25	−178.12 (16)
C19—C12—C13—C18	46.5 (2)		

### Hydrogen-bond geometry (Å, º)

**Table:**

*D*—H···*A*	*D*—H	H···*A*	*D*···*A*	*D*—H···*A*
C12—H12···F1^i^	0.98	2.51	3.402 (2)	151
C5—H5···F1	0.98	2.42	2.833 (2)	105

Symmetry code: (i) *x*, *y*+1, *z*.
